# Hydroxymethanesulfonate from Volcanic Sulfur Dioxide: A “Mineral” Reservoir for Formaldehyde and Other Simple Carbohydrates in Prebiotic Chemistry

**DOI:** 10.1089/ast.2017.1800

**Published:** 2019-03-27

**Authors:** J. Kawai, D. Chris McLendon, H.-J. Kim, S.A. Benner

**Affiliations:** ^1^ Foundation for Applied Molecular Evolution, Alachua, Florida, USA.; ^2^ High Energy Accelerator Research Organization, Institute of Materials Structure Science, Tsukuba, Japan.; ^3^ Firebird Biomolecular Sciences LLC, Alachua, Florida, USA.

**Keywords:** Borate, Organic minerals, Formaldehyde, Sulfur dioxide, Cannizzaro reaction, Hydroxymethanesulfonate, Origins of life, Prebiotic chemistry

## Abstract

While formaldehyde (HCHO) was likely generated in Earth's prebiotic atmosphere by ultraviolet light, electrical discharge, and/or volcano-created lightning, HCHO could not have accumulated in substantial amounts in prebiotic environments, including those needed for prebiotic processes that generate nucleosidic carbohydrates. HCHO at high concentrations in alkaline solutions self-reacts in the Cannizzaro reaction to give methanol and formate, neither having prebiotic value. Here, we explore the possibility that volcanic sulfur dioxide (SO_2_) might have generated a reservoir for Hadean HCHO by a reversible reaction with HCHO to give hydroxymethanesulfonate (HMS). We show that salts of HMS are stable as solids at 90°C and do not react with themselves in solution, even at high (>8 *M*) concentrations. This makes them effective stores of HCHO, since the reverse reaction slowly delivers HCHO back into an environment where it can participate in prebiotically useful reactions. Specifically, we show that in alkaline borate solutions, HCHO derived from HMS allows formation of borate-stabilized carbohydrates as effectively as free HCHO, without losing material to Cannizzaro products. Further, we show that SO_2_ can perform similar roles for glycolaldehyde and glyceraldehyde, two intrinsically unstable carbohydrates that are needed by various models as precursors for RNA building blocks. Zircons from the Hadean show that the Hadean mantle likely provided volcanic SO_2_ at rates at least as great as the rates of atmospheric HCHO generation, making the formation of Hadean HMS essentially unavoidable. Thus, hydroxymethylsulfonate adducts of formaldehyde, glycolaldehyde, and glyceraldehyde, including the less soluble barium, strontium, and calcium salts, are likely candidates for prebiotically useful organic minerals on early Earth.

## 1. Introduction

A recent trend in efforts toward understanding the origin of life seeks models that incorporate geochemical and geophysical features of the early Earth environment into reaction schemes that involve organic molecules (Benner *et al.,* 2010). For example, minerals on early Earth might have acted as catalysts for organic transformations in prebiotic reactions, as potential reactants, and as species that controlled organic reactivity. An example of the first are clays that catalyze organic reactions (Ferris, 2005). For the second, the phosphoborate mineral lüneburgite performs controlled phosphorylation of nucleosides (Kim *et al.,* 2016). For the last, borate minerals were likely useful to control prebiotic reaction sequences to make carbohydrates (Ricardo *et al.,* 2004; Scorei and Cimpoiasu, 2006; Amaral *et al.,* 2008; Šponer, 2008; Cossetti *et al.,* 2010; Pepi *et al.,* 2010; Kim *et al.,* 2011; Kopetzki and Antonietti, 2011; Saladino *et al.,* 2011; Delidovich *et al.,* 2013; Furukawa *et al.,* 2013; da Silva and Holm, 2014).

Further, prebiotic chemistry could have benefitted from organic minerals, solids having defined compositions that contain reduced carbon atoms (as opposed to carbonates). These are scarce on modern Earth, as only the least soluble and most refractory organic minerals (*e.g.,* mellite, Benner *et al.,* 2000) escape rapid consumption on a modern biology-rich Earth. However, on a prebiotic Earth free of life, organic minerals unknown on modern Earth may have been reservoirs for organic species that would otherwise have been too reactive to accumulate in sufficient amounts to be useful for prebiotic synthesis.

Any discussion of prebiotic minerals creates a problem since a mineral cannot be simply a substance of defined composition (*i.e.,* a chemical compound) but must also be *naturally occurring.* To prevent the proliferation of mineral names, the International Mineralogical Association carefully reviews claims of a mineralogical discovery, resolves issues of priority, and certifies the selection of a name. None of this is possible, of course, when discussing prebiotic minerals that can exist on only an abiological world but are rapidly eaten on a biology-rich planet like modern Earth. To reflect this fact, “mineral” is placed within quotation marks when it refers to prebiotic minerals.

For example, Keefe, Miller, Sutherland, and others have suggested that ferrocyanide species might have been prebiotic “mineral” species able to stabilize cyanide, a prebiotically valuable species that self-reacts. This organic “mineral” might have provided nitrogen and carbon resources for prebiotic chemistry (Keefe and Miller, 1995; Patel *et al.,* 2015). Likewise, the Benner group suggested that a branch pentose-borate complex might have been an organic “mineral” reservoir of organic species having the same molecular formula as ribose (Ricardo *et al.,* 2004; Li *et al.,* 2005; Benner *et al.,* 2010, 2012; Kim *et al.,* 2011; Neveu *et al.,* 2013; Furukawa *et al.,* 2015). Indeed, borate-ribose complexes have been productively used in the formation of nucleosides (Becker *et al.,* 2016).

Atmospheric components must, however, be considered as we add geochemistry to models of prebiotic Earth. The Hadean almost certainly had more volcanic activity than today, as radioactive decay and impact energy then was greater than today. Studies of Hadean zircons show that after formation of its iron core, Earth likely had a mantle as oxidizing as today, above the fayalite-magnetite-quartz (FMQ) redox buffer (Trail *et al.,* 2011). This implies that Hadean volcanoes generated large amounts of sulfur dioxide (SO_2_), which in water gives sulfite or bisulfite (HSO_3_^-^, p*K*_a_ ≈ 6.97) anions. SO_2_ can also deliver elemental sulfur (S_8_) and sulfate (SO_4_) by disproportionating in hydrothermal fluids (Kusakabe *et al.,* 2000).

Today, sulfite is oxidized to sulfate in our dioxygen-rich atmosphere. However, a more reducing Hadean atmosphere lacking O_2_ would have allowed bisulfite and its minerals (*e.g.,* hannebachite (CaSO_3_)_2_ ½ H_2_O) (Rai *et al.,* 1991) to accumulate (Marion *et al*., 2013).

Bisulfite reacts with carbonyl-containing compounds to form stable adducts, including with formaldehyde (HCHO), which was likely generated in Earth's prebiotic atmosphere containing water vapor and CO_2_ by the action of ultraviolet light, silent and nonsilent electrical discharge, and volcanic lightning (Cleaves, 2008). HCHO and SO_2_ react to give hydroxymethanesulfonate (HMS = HOCH_2_SO_3_^−^) as an addition product. On modern Earth, HMS is formed in aerosols whenever HCHO and SO_2_ come together (Graedel and Weschler, 1981). The addition is reversible, with an equilibrium constant at pH values above the p*K*_a_ of bisulfite ([SO_3_^−^]_tot_·[HCHO]_tot_/[HOCH_2_SO_3_^−^] ≈ 2 × 10^−4^ *M*) (Sorensen and Andersen, 1970; Boyce and Hoffmann, 1984; Dong and Dasgupta, 1986; Kok *et al.,* 1986). The unimolecular rate constant generating HCHO from HMS at high pH is ∼43 s^−1^ (Sorensen and Andersen, 1970).

Formaldehyde (HCHO) is a key prebiotic precursor for carbohydrates, perhaps best known in the “formose reaction” (Butlerov, 1861; Kim *et al.,* 2011; Appayee and Breslow, 2014). Unfortunately, the formose process requires high concentrations of HCHO under alkaline conditions (pH 10–11) to get started. Under these conditions, HCHO reacts with itself to produce methanol and formate in a process known as the Cannizzaro reaction (Cannizzaro, 1853). Neither of these products has prebiotic value. Further, once HCHO is exhausted, carbohydrates can suffer intercomponent reactions to give complex mixtures that also lack clear prebiotic utility. Particularly easily destroyed are glycolaldehyde and glyceraldehyde, compounds that some models for prebiotic nucleoside synthesis require in large amounts (Powner *et al.,* 2009).

**FIG. 1.**
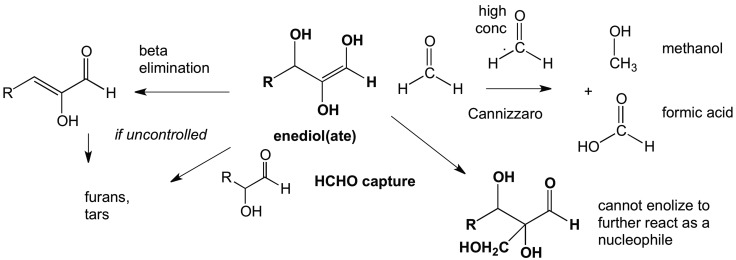
Formaldehyde (HCHO) can capture enediol(ate)s before they react to give complex mixtures of unproductive species. However, at high concentrations, HCHO disproportionates in a bimolecular Cannizzaro reaction to yield unproductive methanol and formate. Both problems can be mitigated if an organic mineral can be found that slowly bleeds HCHO into a prebiotic reaction mixture.

Formaldehyde (HCHO) is especially useful in prebiotic chemistry because of its ability to trap enediolates of carbonyl-containing (C = O) compounds, preventing beta elimination, hydride shift (Appayee and Breslow, 2014), reaction of enediolates with higher carbohydrates, and other processes that give “tars.” At ∼10 m*M* concentrations, HCHO is sufficiently reactive as electrophile to compete with these processes (Fig. 1), even though it exists largely in a hydrated form. For example, in a quantitative study, Kim *et al.* (2011) showed that low concentrations of HCHO would react as the preferred electrophile with any enediol formed by any aldose or ketose in formose-like processes, even though in aqueous solution it is predominately in its hydrated form (H_2_C(OH)_2_). Only when HCHO was consumed did the “yellowing” characteristic of formose processes begin (Ricardo *et al.,* 2006).

Other carbonyl-containing processes are likewise in need of stabilization. For example, glycolaldehyde was undoubtedly produced in the Hadean atmosphere as well, albeit at lower concentrations (Löb, 1913). Glycolaldehyde can act catalytically to fix formaldehyde. This avoids the first, extremely slow, step in the formose process, the (presumed) direct dimerization of HCHO to form glycolaldehyde, something that requires high concentrations of HCHO. Glycolaldehyde reacts readily with itself to form tetroses (Kim *et al.,* 2011). However, if HCHO is present, the enediolate of glycolaldehyde is productively captured to form glyceraldehyde. Likewise, if HCHO is present, the enediolate of glyceraldehyde also can escape tar formation. If borate is present, its binding to adjacent hydroxyl groups (1,2-diols) further controls downstream reactions, diminishing “tar” formation.

These considerations provide relevance to the work reported here. Simple calculations suggest that the amount of Hadean volcanic SO_2_ matched or exceeded the amount of Hadean atmospheric HCHO (Pinto *et al.,* 1980) and, certainly, the amounts of Hadean glycolaldehyde and glyceraldehyde. Thus, the conclusion is inescapable that HCHO generated in the Hadean atmosphere was largely captured on the surface by SO_2_ to give HMS.

**FIG. 2.**
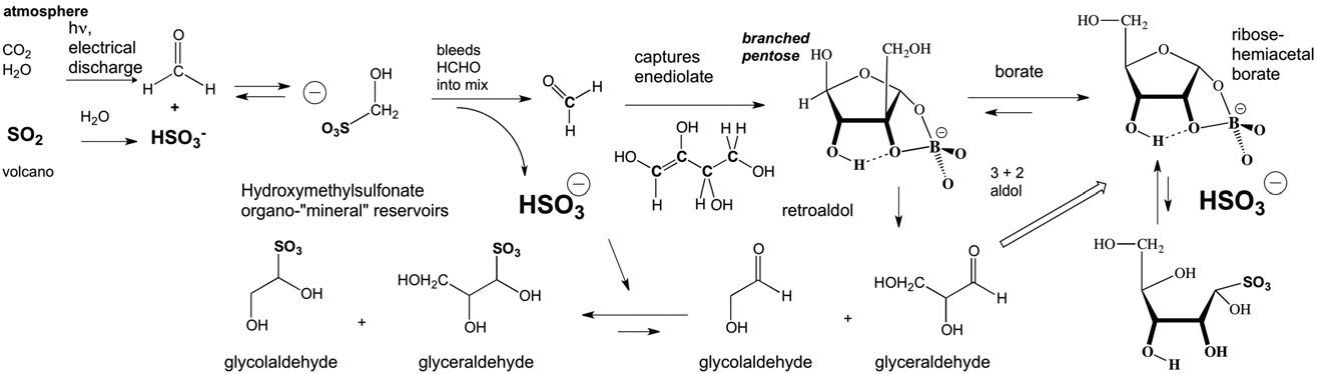
Possible prebiotic reactions involving volcanic SO_2_, HCHO, and other lower carbohydrates. Note how the reaction with ribose involves competition with ring closed forms, here the furanose. Therefore, the equilibrium concentration favors the bisulfite addition product with ribose considerably less than with HCHO, glycolaldehyde, or glyceraldehyde. The same is the case with ketoses. The stereochemistry shown is arbitrary.

This raises questions. First, we might ask whether this is “bad” for prebiotic chemistry, because it removes HCHO needed in prebiotic schemes. Or is it “good,” because HMS can accumulate and, because of the reversibility of the reaction that forms it, provide a continuous source of HCHO, which would otherwise be unavailable for prebiotic chemistry (Fig. 2)? Is it possible that the reverse dissociation to give HCHO and bisulfite would give HCHO in an aquifer at high enough concentrations to control undesirable enediolate reactivities, but not so high as to waste material in the Cannizzaro reaction, whose rate scales with the square of HCHO concentration? Last, can bisulfite also provide stable reservoirs of higher carbohydrates, such as glycolaldehyde and glyceraldehyde?

We report here a study of this reaction manifold using the sodium salt of ^13^C-labeled HMS. Dihydroxyacetone (DHA) was used as a model “lower” carbohydrate in sodium carbonate-bicarbonate buffers at pH 10.5, reasonable from serpentinizing basalts. We show that HMS, by slowly dissociating to give free HCHO, can capture the enediolate of DHA before it reacts with itself to generate 4-hydroxymethyl-1,3,4,5-tetrahydroxy pentane-2-one. We then show how bisulfite might react reversibly with other unstable carbohydrates to provide reservoirs useful in prebiotic synthesis.

## 2. Materials and Experimental Methods

### 2.1. Materials

Materials used were deuterium oxide (D_2_O; Acros), chloroform-D (CDCl_3_; Cambridge Isotope Laboratories), sodium deuteroxide (NaOD; Acros Organic), hydroxylmethyl sulfonate (CH_3_NaO_4_S; Aldrich), dihydroxyacetone (C_3_H_6_O_3_; Aldrich), glycolaldehyde (C_2_H_4_O_2_; 0.0476 *M,* Omicron, C-13 labeled), sodium bisulfite (NaHSO_3_; Fisher), paraformaldehyde (HO-(CH_2_O)_*n*_ H, C-13 labeled; Sigma), erythrulose (C_4_H_8_O_4_; Sigma-Aldrich), glyceraldehyde (C_3_H_6_O_3_; Sigma), boric acid (Sigma-Aldrich), and sodium carbonate (Fisher).

### 2.2. Experimental methods

#### 2.2.1. Synthesis of ^13^C-labeled sodium hydroxymethanesulfonate

^13^C-Labeled paraformaldehyde (30 mg, 1 mmol, Sigma) was treated with sodium bisulfite (1 mmol, 104 mg, Fisher) in deionized water (1 mL) at 65°C. The powder did not immediately dissolve, so the pH of the mixture was slowly raised by dropwise adding 1 *M* NaOH (Fisher) until the solution became clear. The mixture was then lyophilized to obtain the sodium salt of HMS, which gave a ^13^C NMR signal at 74.51 ppm (CH_3_OH standard at 49.5 ppm, 75 MHz, room temperature). Neither methanol nor formic acid, the Cannizzaro products, were seen.

#### 2.2.2. Reaction of ^13^C-labeled sodium hydroxymethanesulfonate with dihydroxyacetone

A sample of the DHA (0.5 mmol), which gives the same reactive enediolate as glyceraldehyde, was dissolved in borate-carbonate buffer (1.1 *M* Na_2_CO_3_, 0.278 *M* H_3_BO_3_, 1 mL D_2_O, pH ≈10.5, all at 25°C) with ^13^C-labeled HMS (1.0 mmol). A parallel reaction was run without HMS but with an equivalent amount of ^13^C-labeled formaldehyde (1.0 mmol). The reaction mixture samples were incubated at 65°C and room temperature, respectively, for 24 h. The samples were then directly analyzed by ^13^C NMR spectroscopy at room temperature.

#### 2.2.3. Parallel large-scale reaction of unlabeled HMS with DHA

A mixture of DHA (0.90 g, 10 mmol) and unlabeled HMS (4.10 g, 30 mol, 3 fold excess) was dissolved in degassed borate-carbonate buffer (200 mL H_2_O, 1.1 *M* Na_2_CO_3_, 0.278 *M* H_3_BO_3_, pH ≈ 10.5, all at 25°C). This mixture was then incubated with stirring at 65°C for 2 days and at room temperature for 10 days under an N_2_ atmosphere.

To prepare for proton NMR analysis, the mixture was neutralized by ion exchange resin (Dowex-50, H^+^ form, Acros), which released the carbonate as carbon dioxide. The resin was then removed by filtration and the filtrate lyophilized. The borate ions were then removed by addition of CH_3_OH (100 mL), followed by evaporation of the trimethylborate formed by rotary evaporation to give a reddish-brown solid. The samples were dried further overnight under high vacuum and analyzed by NMR.

#### 2.2.4. Derivatization of the carbohydrate products

For further analysis, portions of the residues were dissolved in pyridine (100 mL) containing dimethylaminopyridine (250 mg), treated with acetic anhydride (15 mL), and incubated at room temperature (1 day). Solvents were removed by rotary evaporation, the residue was treated with aqueous 1 *M* HCl at room temperature, and water was added. Acetates were extracted with CH_2_Cl_2_, and the aqueous phase containing pyridinium hydrochloride was discarded. The CH_2_Cl_2_ was removed by rotary evaporation to give a reddish residue. The products were separated by silica gel column chromatography (ethyl acetate:hexane gradient = 1:2 to 2:1; Fluka silica gel, 230–400 mesh), and the effluent was collected in fractions. Each fraction was evaporated, and the residue was weighed to estimate yields and characterized by ^1^H NMR. To identify each fraction, standard materials were used as comparison.

#### 2.2.5. Mass spectral analysis

The products of the reactions between unlabeled HMS and unlabeled HCHO were analyzed before acetate derivatization (but after removing carbonate and borate) by mass spectrometry (Agilent 6220 ESI-TOF with Agilent 1100 LC; Mass Spectrometry Services, University of Florida) using electrospray ionization (ESI) and time-of-flight (TOF) analysis. The samples were prepared as a solution of acidified methanol (0.1% formic acid); injecting of 5 μL of sample into the electrospray source was done at a rate of 200 mL/min. ESI settings were as follows: voltage 4000 V, source temperature 350°C, and cone voltage of 60 V. The TOF analyzer was scanned over *m/z* 30–3200 with a 2 s integration time (data not shown).

## 3. Results

### 3.1. Comparing the ^13^C spectra of the product manifolds obtained by reaction of dihydroxyacetone with ^13^C-formaldehyde (HCHO) directly, or with (H^13^CHO) delivered *in situ* from ^13^C-hydroxymethanesulfonate

Nuclear magnetic resonance was first used to test the hypothesis that HMS can replace formaldehyde (HCHO) in reactions that may simulate the formation of carbohydrates in borate-containing effluents emerging from serpentinizing igneous basalts. This reaction has previously been studied in quantitative detail (Kim *et al.,* 2011; Neveu *et al.,* 2013), where it was shown that DHA or glyceraldehyde enolize in sodium carbonate-borate buffer (pH ≈ 10.5) to give a common enediolate. This enediolate reacts with HCHO to give erythrulose, which, under guidance from borate, enolizes to give another enediolate. This reacts with a second molecule of HCHO to give either a branched tetrose (2-hydroxymethyl-2,3 dihydroxypropanal) or erythrulose. The branched tetrose has no enolizable protons but suffers a crossed-Cannizzaro reduction with HCHO to give 2-hydroxymethyl-1,2,3 trihydroxypropane, a dead-end product.

Erythrulose, as the principal product, can enolize, however. The resulting enediolate was shown by Kim *et al.* (2011) to react at its less hindered center to give a mixture of erythro and threo stereoisomers of branched pentoses (C5). The ^13^C NMR spectra of these species arising from H^13^CHO is characteristic of the product ratio, especially in the collection of NMR signals at 60–65 ppm (MeOH standard at 49.5).

**FIG. 3.**
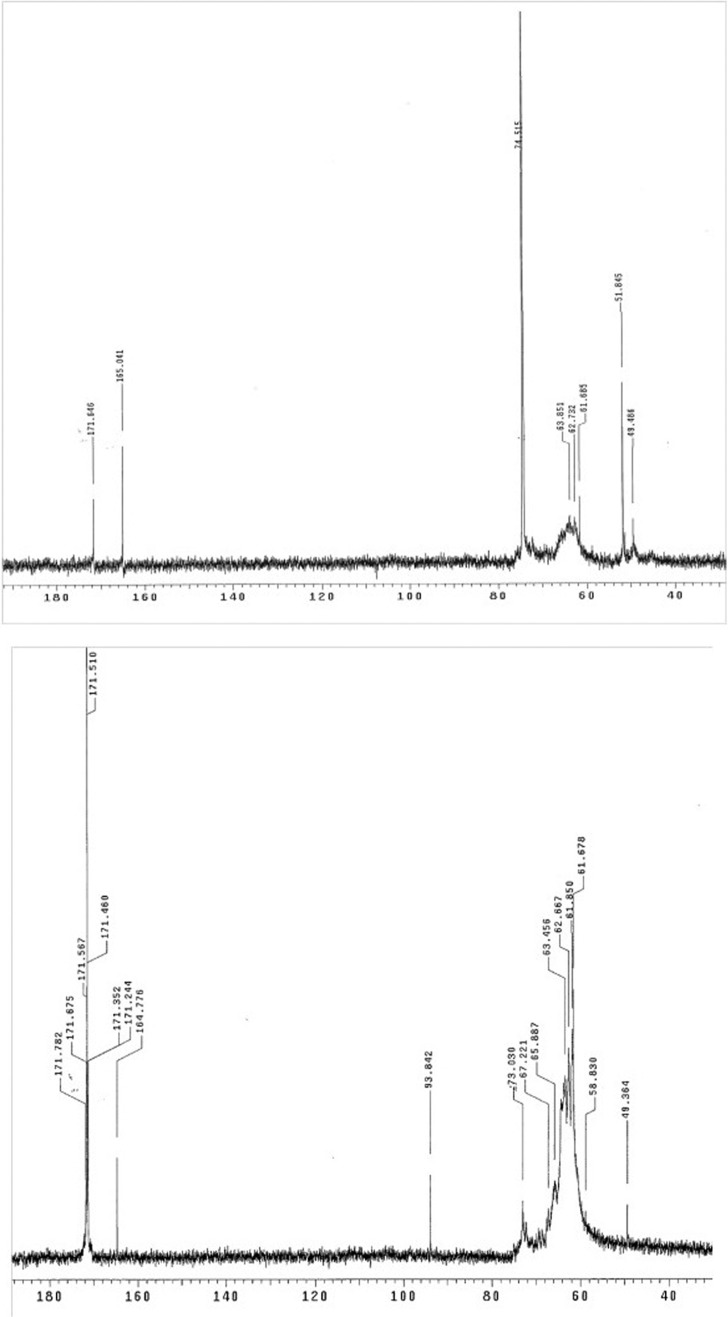
Comparison by ^13^C NMR spectroscopy of the reaction of DHA with H^13^CHO “bled” into the reaction mixture from ^13^C-labeled HMS (top, signal at ∼74.5) or H^13^CHO added directly (bottom). ^13^C-HMS resonates at 74.2 ppm. Reactions were run in NaCO_3_ (1.1 *M* all at 65°C) H_3_BO_3_ (0.278 *M*) buffer at pH 10.4. Note that the manifold of peaks arising from ^13^CH_2_OH units in the branched pentoses at 60–65 ppm are essentially the same. Also note the presence of substantially more HCOOH (171 ppm, ionized to give formate) from the Cannizzaro reaction when free H^13^CHO is added directly. MeOH (49.5) was added as an internal standard. The pH-dependent signal from carbonate buffer is at ∼165 ppm.

The Kim *et al.* (2011) experiment was repeated, and repeated in parallel where H^13^CHO was replaced by ^13^C-HMS. The NMR spectra are strikingly similar (Fig. 3). This indicates that H^13^CHO added to solution directly can be replaced by H^13^CHO “bled” into the medium by the unimolecular decomposition of HMS. The similarity of the manifolds of ^13^C signals showed that the product ratios were quantitatively identical, regardless of the source of the H^13^CHO. Further, unlike when H^13^CHO was used, very few Cannizzaro products (methanol at 49.5 ppm and formic acid at ∼171 ppm) were seen. This suggested that the concentration of H^13^CHO bled from HMS was never high enough to allow this reaction (bimolecular in HCHO) to occur to a substantial degree.

### 3.2. Stability of sodium hydroxymethanesulfonate

To estimate the thermal stability of salts of HMS, a sample of sodium HMS (6.55 g) was weighed and then heated at 90°C. The weights of the material were measured over a 24 h period. After 24 h, ∼3.5% loss of mass was observed, entirely due to dehydration. Thus, this form of the organic “mineral” was stable to thermal degradation under these conditions.

### 3.3. Solubility of hydroxymethanesulfonate minerals

Aliquots (1.0 mL) of a saturated solution of sodium HMS (∼0.95 g/mL) were treated with aliquots (0.25 mL) of 1 *M* solutions of the chlorides of barium, strontium, calcium, and magnesium as their chloride salts (all at 25°C). Precipitates were observed for the first three divalent cations, but not with magnesium. The barium HMS was the least soluble of the three. A sample of this was prepared, 1 g was suspended in pure water (at 25°C), a 200 μL sample was withdrawn, and the residue was weighed to give a solubility of 386 g/L. In parallel, no precipitation was seen with any transition metal tested, including manganese, iron, cobalt, nickel, copper, or zinc, all as divalent cations.

### 3.4. Reaction of sulfite with glycolaldehyde, glyceraldehyde, dihydroxyacetone, and ribose

**FIG. 4.**
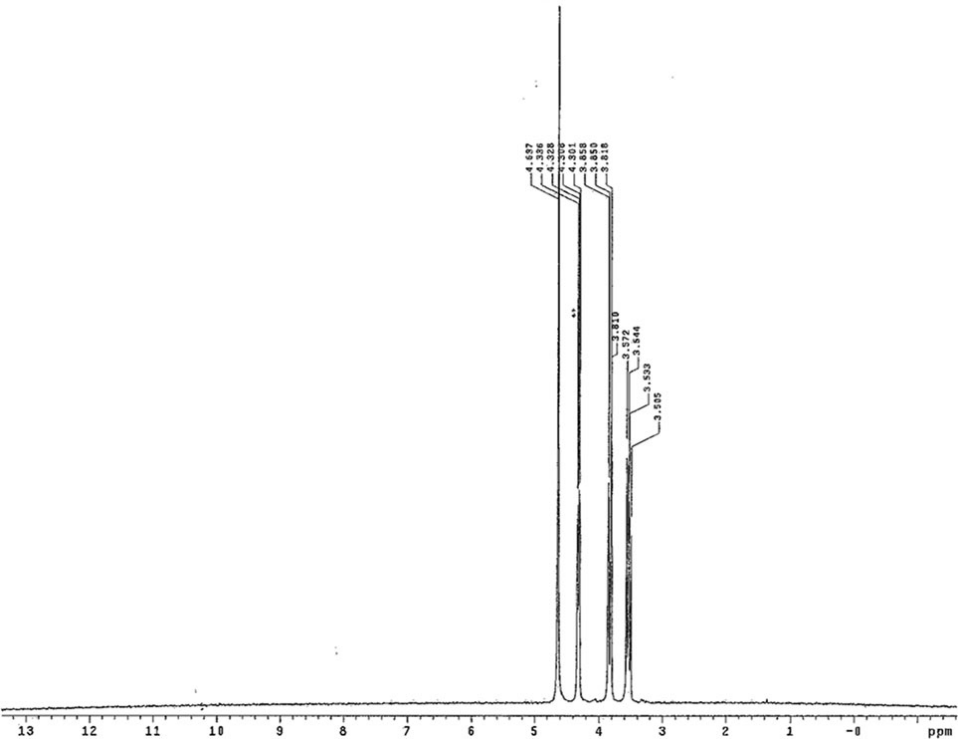
^1^H NMR spectra of 1:1 mixture of glycolaldehyde and sodium bisulfite, obtained in D_2_O at 25°C. Glycolaldehyde has proton resonances at 4.9 ppm (triplet, C1 of the hydrate) and 3.3 ppm (doublet, C2 of the hydrate, data not shown). Both are missing in the spectrum, which has new resonances at 4.3 and 3.85 ppm (C1, adduct, split by C2 proton, two diastereomers) and 3.5 ppm (C2). Notice also the coupling due to the new stereogenenic center. Absence of detectable amounts of glycolaldehyde suggests that adduct had consumed more than 99% of the substrate.

**FIG. 5.**
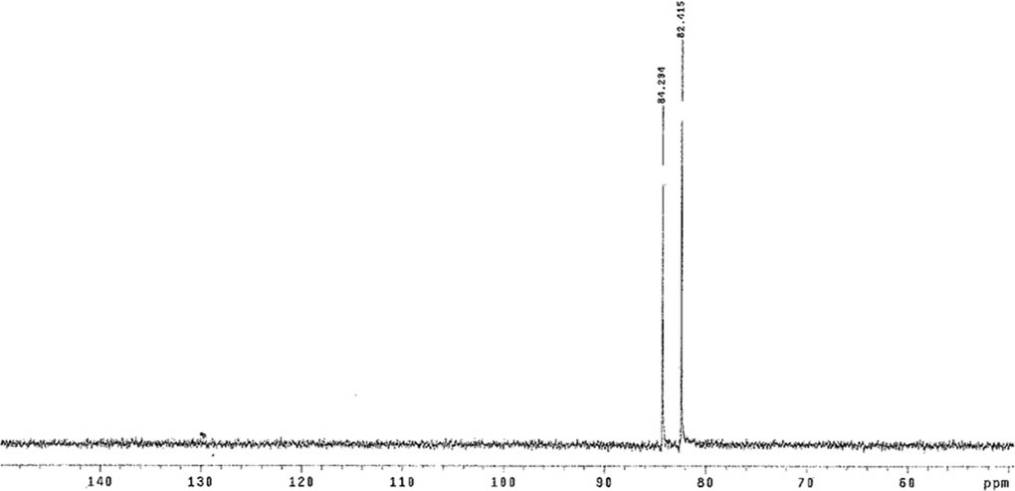
^13^C NMR spectra of a 1:1 mixture of 1-^13^C-labeled D,L-glyceraldehyde and sodium bisulfite, obtained in D_2_O at a pH of 4.76 at 25°C. The two resonances (84.3 and 82.4) are assigned to be the two diastereomeric adducts in a ratio of ∼47:53. Glyceraldehyde itself had a single resonance at 89.95 ppm (data not shown), which is assigned to the hydrated species. Again, undetectable of a resonance at 89.95 ppm suggests that adduct had consumed more than 99% of the substrate.

We then determined whether other prebiotically useful carbonyl compounds that are unstable, especially with respect to self-reaction, might be stabilized as bisulfite addition products. The ^1^H NMR spectrum of a 1:1 mixture of glycolaldehyde and sodium bisulfite was seen to contain exclusively the bisulfite adduct (Fig. 4, all at 25°C). These data indicate an equilibrium constant strongly favoring adduct formation. The same conclusion was compelled by the ^13^C NMR spectrum of a 1:1 mixture of ^13^C^1^-labeled glyceraldehyde and sodium bisulfite in D_2_O (Fig. 5).

**FIG. 6.**
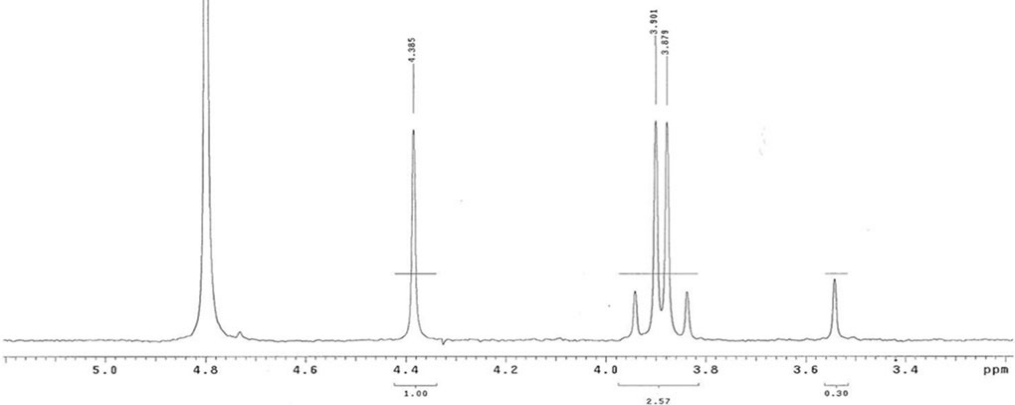
^1^H NMR spectra of DHA plus sodium bisulfite (in a 1:1 mixture, 10 m*M* each), obtained in D_2_O at 25°C. DHA alone has a proton resonance of 4.38 (with water set at 4.8 ppm; the peak at 3.7 is the hydrate of DHA). Here, the presence of free and adducted DHA allows an equilibrium constant to be calculated, at ≈1.6 × 10^-3^M. The integral shows that the (2.57 = integral for total adduct protons, versus 1.3 = integral for total DHA proton, or 2:1 adduct:free DNA) yields an equilibrium constant [DHA][bisulfite]/[adduct] = 1.6 × 10^−3^ *M*.

Dihydroxyacetone (DHA) also forms an adduct with bisulfite. Here, however, adduct formation was not complete with equal amounts of DHA and bisulfite (10 m*M* each). Instead, the ratio of the bisulfite adduct to the free DHA was approximately 2:1 (Fig. 6). This allowed ready calculation of an equilibrium constant of 1.6 × 10^−3^ *M*. This incomplete formation of the adduct is consistent with a model where steric hindrance opposes addition of nucleophiles to the central carbonyl group, and is reflected as well in the smaller amount of acetone hydrate compared to acetaldehyde hydrate in equilibrium.

**FIG. 7.**
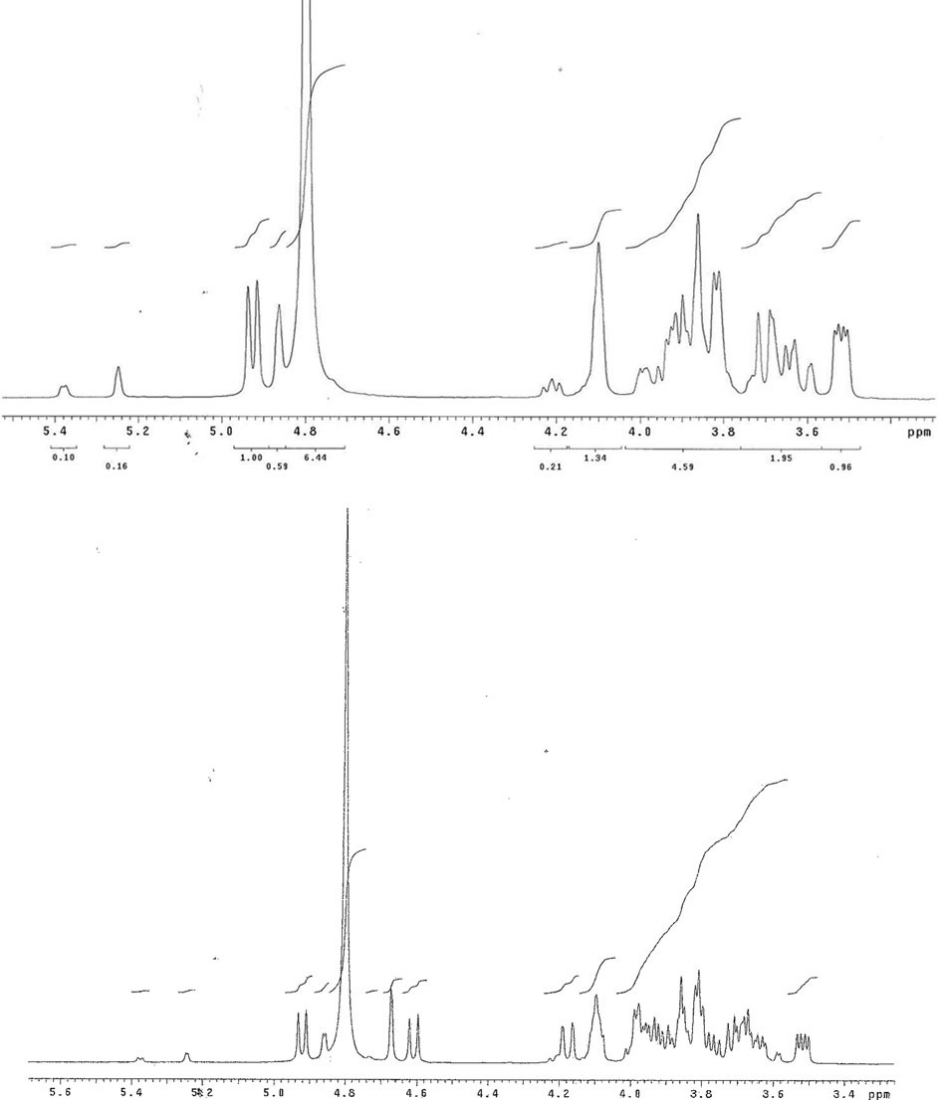
^1^H NMR spectra of ribose (top) and ribose plus sodium bisulfite (a 1:1 mixture, 10 m*M* each), obtained in D_2_O (set at 4.8 ppm) at 25°C. Attention is drawn to the peaks at 4 4.0–4.92, 5.25, and 5.38, which are the alpha and beta isomers of the furanose and pyranose forms. The new resonances at 4.6 (doublet) and 4.67 (broad) are the bisulfite addition products (C1). Note that these represent perhaps only 20–30%% of the total. The estimated equilibrium constant [ribose][bisulfite]/[adduct] ≈ 2 × 10^−2^ *M*.

Finally, the ability of ribose to form a bisulfite adduct was estimated by ^1^H NMR in D_2_O containing bisulfite (10 m*M*) (Fig. 7). As with DHA, a substantial amount of the carbonyl compound remains. The precise amount was difficult to estimate, as ribose itself has four forms in equilibrium (alpha and beta forms of the furanose and pyranose), each giving different NMR signals. However, 1 × 10^−3^ *M* is not a bad estimate for the equilibrium constant for the addition of bisulfite to ribose. Here, although ribose nominally has an aldehyde, bisulfite addition must compete with cyclization to form the closed furanose and pyranose rings.

## 4. Discussion

Subareal volcanism from today's Earth generates *ca.* 2 × 10^11^ mol/year of sulfur dioxide (SO_2_) (Andres and Kasgnoc, 1998). If we assume that the Hadean mantle had similar oxygen fugacity at the relevant time (Trail *et al.,* 2011), this likely represents a lower limit on the rate of SO_2_ production during the period relevant to prebiotic synthesis. This is similar to the classical estimate of 10^11^mol/year for the annual Hadean production of HCHO (Pinto *et al.,* 1980).

Thus, it seems reasonable to assume that most HCHO produced in the Hadean atmosphere ended up in the hydrosphere as HMS. The HMS may have been formed in aerosol droplets in the atmosphere itself, but certainly would have occurred in the hydrosphere, capturing HCHO and SO_2_ that rained into it. Only if the amount of Hadean volcanism is severely overestimated or the rate of Hadean HCHO formation severely underestimated should we expect free HCHO in large amounts to serve as a prebiotic reagent.

The results reported here show that these estimates do not preclude HCHO as a prebiotic precursor. Via its dissociation reaction, HMS is shown by these experiments to be suitable as a continuous source of HCHO at concentrations high enough to participate in various prebiotic processes, here, the borate-moderated formose process that generates 5-carbon carbohydrates from glycolaldehyde (Kim *et al.,* 2011). Here, bleeding of HCHO into an aqueous environment is high enough to trap enediolates formed from carbohydrates produced at pH values likely to be obtained in serpentinizing rocks, preventing them from suffering a range of destructive reactions. The key result here is the experimental demonstration that the ratio of products in the borate-moderate formose reaction, judged by ^13^C labeling, is the same whether HCHO is added directly or is bled into the reaction via dissociation of HMS. These products are mainly erythro- and threo-branched pentoses (Kim *et al.,* 2011).

As Orgel pointed out some time ago (Orgel, 2004), substantial concentrations of HCHO did not likely accumulate on a Hadean Earth in any case. At the alkaline conditions expected from serpentinization, high concentrations of HCHO are destroyed by the Cannizzaro reaction to give methanol and formate, both essentially useless as prebiotic precursors. The results reported here show that if HCHO is bled (slowly released) from a HMS reservoir, its steady-state concentration is insufficient to support the Cannizzaro reaction.

The effectiveness of enediolate trapping depends on the rate of formation of the enediolate compared to the rate of formation of HCHO by dissociation of HMS, which in turn depend on the relevant reaction rate constants and the concentrations of the species involved. The rate constant for cleavage of HMS is 43 s^−1^ (Sorensen and Andersen, 1970), at least 2 orders of magnitude higher than the rate constant for the enolization of glyceraldehyde at pH 10.5 (Kim *et al.,* 2011), a reasonable pH for serpentinizing basalts. Thus, efficient capture of enediolates by HCHO generated from HMS is likely in a prebiotic scenario that incorporates both chemistry and geology.

Of course, the high water solubility of the sodium salt of HMS (∼1 g/mL at 25°C) means that it could accumulate in an arid desert environment only. However, the calcium salt, the strontium salt, and especially the barium salt of HMS are rather insoluble in water. Direct experiments showed that the barium bis(hydroxymethanesulfonate) (BaC_2_H_6_S_2_O_8_, assuming no hydration) has a solubility in water at 25°C of 386 g/L, essentially the same as that of halite (the mineral form of NaCl). These “minerals” are as yet unknown. Certainly on contemporary Earth, with its panversal biosphere, any such HMS minerals would be rapidly consumed by microorganisms.

Interestingly, bisulfite derived from volcanic SO_2_ appears able to perform the same role for Hadean atmospheric glycolaldehyde (Löb, 1913) and glyceraldehyde. The addition of bisulfite to these alpha-hydroxy aldehydes, while also reversible, appears to go largely to completion with an equilibrium constant of less than ∼10^−5^ *M*. Glycolaldehyde and glyceraldehyde in substantial concentrations are invoked in several models for the Hadean origin of ribonucleoside derivatives, including those of Powner *et al.* (2009). While accumulations of large amounts of *free* glycolaldehyde and free glyceraldehyde required by these models seems unlikely due to their self-reaction, these models may now be revisited where the precursors are the bisulfite addition products of glycolaldehyde and glyceraldehyde.

The bisulfite addition products of DHA (where the C═O unit is in the form of a ketone) and ribose are thermodynamically less stable. For the first, this is understood under a steric model. Just as the hydrate of DHA is less stable than the hydrate of glyceraldehyde, so is its bisulfite addition product. The relatively little bisulfite addition product formed by ribose is understood by its need to compete with the formation of cyclic forms of this pentose. In the presence of borate, this competition is even less favorable.

This hypothesis has implications for the mineralogy of early Earth. As noted in the introduction, sulfur (IV) on contemporary Earth is readily oxidized in the dioxygen-containing atmosphere. Therefore, while sulfate minerals are widespread today on Earth, sulfite minerals are not. Exceptions include calcium sulfite (hannebachite, soluble at 25°C to 0.043 g/L, less than the solubility of calcium sulfate dihydrate, which is the evaporate mineral gypsum), scotlandite (lead sulfite), gravegliaite (manganese sulfite), and orschallite (calcium sulfate-sulfite) (Weidenthaler *et al.,* 1993). The interaction of aldehyde on the surface of sulfite minerals led to the adduct formation (Pitsch *et al.,* 2000). Thus, this work should stimulate this overlooked area of mineralogy and help guide the exploration of other rocky planets.

## Notes

Associate Editor: David Deamer

## Abbreviations Used

DHA: dihydroxyacetone
ESI: electrospray ionization
HMS: hydroxymethanesulfonate
TOF: time-of-flight
